# International Law, COVID-19 and Feminist Engagement with the United Nations Security Council: The End of the Affair?

**DOI:** 10.1007/s10691-020-09440-4

**Published:** 2020-10-31

**Authors:** Catherine O’Rourke

**Affiliations:** School of Law, Ulster University, Jordanstown, BT37 0QB UK

**Keywords:** International Law, COVID-19, United Nations Security Council, Crisis

## Abstract

The gendered implications of COVID-19, in particular in terms of gender-based violence and the gendered division of care work, have secured some prominence, and ignited discussion about prospects for a ‘feminist recovery’. In international law terms, feminist calls for a response to the pandemic have privileged the United Nations Security Council (UNSC), conditioned—I argue—by two decades of the pursuit of the Women, Peace and Security (WPS) agenda through the UNSC. The deficiencies of the UNSC response, as characterised by the Resolution 2532 adopted to address the pandemic, manifest yet again the identified deficiencies of the WPS agenda at the UNSC, namely fragmentation, securitisation, efficacy and legitimacy. What Resolution 2532 does bring, however, is new clarity about the underlying reasons for the repeated and enduring nature of these deficiencies at the UNSC. Specifically, the COVID-19 ‘crisis’ is powerful in exposing the deficiencies of the crisis framework in which the UNSC operates. My reflections draw on insights from Hilary Charlesworth’s seminal contribution ‘International Law: A Discipline of Crisis’ to argue that, instead of conceding the ‘crisis’ framework to the pandemic by prioritising the UNSC, a ‘feminist recovery’ must instead follow Charlesworth’s exhortation to refocus on an international law of the everyday.

In this brief reflection, I want to suggest that the dynamics around COVID-19, specifically the delay and inaction of the United Nations Security Council (hereafter ‘Security Council’) in responding to the pandemic, may have longer term implications for feminist engagement with international law. Thus, the way that much feminist engagement tends to privilege the Security Council within global governance may be coming to an end (Otto 2009).

The gendered implications of COVID-19, most notably in terms of increased exposure to gender-based violence (see for example, Neetu et al. 2020) and the gendered division of increased care work (see for example, Power 2020), have secured considerable prominence in public debate and in global governance discussions about the pandemic. Indeed, if we look at the United Nations Secretary-General (hereafter ‘Secretary-General’), he accompanied his call for a global ceasefire^1^ with a call to end gender-based violence and called on governments to ensure peace at homes around the world and to put women’s safety first as they respond to the pandemic.^2^ The centrality of gender relations does evidence some success of feminist messages in international law and global governance. Indeed this prominence has, more optimistically, led to calls for a feminist recovery from the pandemic.^3^ As someone who works primarily on gender and conflict in international law, what has been interesting to me about feminist responses and engagement around the pandemic and international law is this central positioning of the Security Council in many of those feminist calls for response and change. For example, Madeleine Rees, the Secretary-General of Women’s International League for Peace and Freedom, has written a widely circulated call on the Security Council to ‘do something’: “What on earth is the Security Council doing?”, she asks.^4^ The NGO Working Group on Women, Peace and Security issued a joint statement calling on the Security Council for a resolution to address the pandemic.^5^ The Group of Women Leaders Voices for Change and Inclusion—which includes a number of prominent international women leaders such as Helen Clark, Margot Wallström, Navi Pillay and others—wrote an open letter to the Security Council President again calling for a resolution on the pandemic.^6^

What manifold feminist calls to action from the Security Council reveal, first, is the central position of the Security Council in feminist engagement with international law. And, of course, that impulse to look to the Security Council is not new with the pandemic. Rather, that impulse is grounded in over two decades of feminist prioritisation of the Security Council as the institution best placed to advance women’s rights or participation in an insecure world (see generally, Cockburn 2007; Anderlini 2007). That dynamic is epitomised by campaigns for Security Council Resolution 1325 and subsequent resolutions dealing with this Women, Peace and Security agenda,^7^ that marks its 20th anniversary this year. Admittedly, feminist engagement with the Security Council has also long been characterised by ambivalence. This ambivalence is variously grounded in suspicion of its inherently militarist and selective composition and function (see for example, Otto 2009); the legitimacy deficits that result (see for example, Otto 2010); sober assessment about limited material gains from engagement to date^8^; as well as more fundamental questioning of the so-called ‘will to power’ of feminist engagement with institutions such as the Security Council (see generally, Halley et al. 2019; Otto 2019). Nevertheless, that this multifaceted ambivalence has not previously grounded a feminist rejection of the institution says much about the enduring place of the Security Council in feminist strategy in international law (O’Rourke 2017). Second, interestingly, the turn to the Security Council to respond to the pandemic also reveals how the Security Council’s inaction—principally the failure and delay around adopting a resolution—is read by many feminist actors as an unqualified failure of the Security Council to meet its mandate to respond to threats to international peace and security.

We now have a resolution: Resolution 2532 was adopted on 1 July 2020 by the Security Council to respond to the pandemic.^9^ Resolution 2532 reiterates the Secretary-General’s call for a global ceasefire in response to the pandemic: “a durable humanitarian pause for at least 90 consecutive days”, in order to enable delivery of humanitarian assistance,^10^ but then establishes an exception to this call for military operations against ISIL, Al Qaeda, Al Nusra Front and associated groups, as well as all terrorist groups so designated by the Security Council.^11^ The resolution requests the Secretary-General to help ensure that all relevant parts of the UN, including UN country teams, accelerate their response to the pandemic^12^; and to update the Security Council on such efforts, in particular in terms of any impact on the ability of peacekeeping operations and Special Political Missions mandated by the Security Council to discharge their mandates.^13^ With further regard to peacekeeping operations, the Secretary-General is asked to instruct peacekeeping operations to support host country authorities to ensure humanitarian access to internally displaced persons (IDPs) and refugees; and for the Secretary-General and member states to take all appropriate steps to protect the safety, security and health of all UN personnel in UN peace operations, while maintaining the continuity of such operations.^14^ In the final substantive operative paragraph, the resolution:

*Acknowledges* the critical role that women are playing in COVID-19 response efforts, as well as the disproportionate negative impact of the pandemic, notably the socio-economic impact, on women and girls, children, refugees, internally displaced persons, older persons and persons with disabilities, and *calls* for concrete actions to minimize this impact and ensure the full, equal and meaningful participation of women and youth in the development and implementation of an adequate and sustainable response to the pandemic;^15^

I argue that the deficiencies of Resolution 2532, although certainly not about feminist engagement, have sharpened dilemmas that are confronted by feminist engagement with international law and especially the Security Council. These are dilemmas that have been present for some time. For example, the resolution and its failure to even reference the World Health Organization (WHO)—in contrast to the resolution on COVID-19 of the UN General Assembly^16^—even in the preamble, does raise questions about the purpose of the resolution. To borrow from Benvenisti and Downs (2017): is Resolution 2532 a type of ‘fragmentation strategy’, whereby the institution that actually has resources and authority to lead a global response to the pandemic is distracted from by the Security Council, including the displacement of attention and resources from the WHO to the Security Council? There is also a question of whether the Security Council advances its mission creep beyond threats to international peace and security with this resolution (Otto 2009). Further, does the Security Council adopting a resolution around a pandemic reflect the securitisation of a public health issue (O’Rourke and Swaine 2018, 171)?

Even assessing the resolution on the narrowest possible feminist terms, namely reference to women and the WPS agenda, the results are doubtful. Whilst Resolution 2532 does—in its final operative paragraph—reference women,^17^ the resolution does not reference the WPS resolutions at all. In that sense, therefore, the Security Council has opted not to endorse its existing normative commitments around gender equality, nor to activate its infrastructure to advance those normative commitments through the resolution. Ultimately, the weaknesses of the resolution mean that it is largely rhetorical. Resolution 2532 thereby reaffirms suspicion that the adoption of the resolution was really—rather than being about providing leadership for a global response to the pandemic—in fact about the Security Council and its legitimacy and being seen to do something to justify its existence and activities.

These problems with the Security Council as manifested through Resolution 2532 –fragmentation, securitisation, efficacy and legitimacy—are familiar problems in feminist engagement with the Security Council. In fact, as noted above, these problems reflect how much feminist analysis characterises the WPS resolutions (see generally, Davies and True 2019). What Resolution 2532 does arguably bring, however, is new clarity about the underlying reasons for the repeated and enduring nature of these problems at the Security Council. Specifically, the COVID-19 ‘crisis’ is powerful in exposing the deficiencies of the crisis framework in which the Security Council operates. Critique of international law’s crisis tendency is not new. It was articulated most forcefully in 2002 by Hilary Charlesworth in Modern Law Review in her seminal article, ‘International Law: A Discipline of Crisis.’ The article’s central argument is that the focus of international lawyers on crisis shields the discipline from more fundamental questions and inquiries (Charlesworth 2002). This is a critique that has acquired further powerful resonance in light of the pandemic.

To reprise Charlesworth’s (2002, 382) key arguments: she talks about the contentious construction of crisis and the ethical costs of crises. In terms of the contentious construction of crisis, she talks about the negotiability of facts. Therefore, the crisis model in international law tends to assume that elements of the crisis are ‘facts’ that are uncontroversial and ripe for picking. But of course, any critical reading of international law will reveal that that is not the case. For example, in the UK, tabloid newspaper headlines talked about the ‘first’ lockdown killings, referring to women who are killed by violent male partners under lockdown. However, domestic homicide is a tragically familiar occurrence: two women a week die in the UK at the hands of male partners.^18^ The primary framing of such killings as a consequence of the pandemic obscures their roots in gendered violence and inequality.

Next, Charlesworth (2002, 384) talks about the lack of analytical progress due to the ‘crisis’ framing of international law. By treating the crisis as ‘new’, we rediscover issues constantly and analyse them without building on past scholarship or learning. To illustrate, COVID-19 is characterised as a new problem, rather than a manifestation of, for example, entrenched capitalist development models and longstanding indiscriminate damage to the natural world. Charlesworth (2002, 384) also talks about the problem of ‘thin description.’ Thus, the crisis model leads us to concentrate on a single event or series of events and to often miss the larger picture. Therefore the core ‘problem’ of the pandemic becomes about the shortage of Personal Protective Equipment or China’s lack of disclosure, rather than the long-running immiseration of public services and the social state that the pandemic has exposed.

Further, in terms of ‘the ethical cost of crisis,’ the focus on crisis narrows the agenda of international law, according to Charlesworth (2002, 386). Therefore, the only possible course of action in the face of crisis is to act, or not to act: either the Security Council adopts a resolution or it does not. But of course, there is so much between those two poles. In addition, this narrow agenda—in which the Security Council either adopts a resolution or fails to adopt a resolution—shields the ultimate resolution from critique. Further, the characterisation of crises and the Security Council role means that, whilst the Security Council can mandate its country missions to account for COVID-19,^19^ the Security Council cannot address any of the structural underpinning problems of the pandemic and its causes. Finally, Charlesworth (2002, 388) condemns the silencing effect of the crisis and how the crisis is silencing many other voices and priorities. Consider, for example, what China is attempting to do in Hong Kong under the cover of the pandemic.^20^ Ultimately, whilst feminist prioritisation of the Security Council for two decades plus has preconditioned and predetermined the approach to the Security Council for COVID-19, this feminist engagement further privileges the Security Council in international law and concedes the crisis characterisation of the pandemic.

As we are learning, the crisis characterisation is, in fact, antithetical to a feminist recovery. And here, instead of looking to international actors, I look locally to Belfast where women’s organisations and feminist actors have developed a truly extraordinary ‘Feminist Recovery Plan’ for the pandemic.^21^ This initiative, rather than emphasising the crisis and discontinuity caused by COVID-19, instead emphasises the social and economic precarity that characterised the pre-COVID-19 world and that has predetermined the gendered vulnerabilities to which international organisations are now calling attention. The Plan defines a feminist recovery accordingly:

This plan will use a mix of political and economic policy-making recommendations to advocate for a feminist recovery to COVID-19 with the aim of not only avoiding deepening gender inequalities through recovery planning, but also tackling the gendered inequalities that already exist in our society.^22^

This framing is in stark contrast to the dominant approach of international law, particularly as imagined by the Security Council. As the Feminist Recovery Plan makes clear, individuals and families—and yes, many women—were in ‘crisis’ long before this pandemic hit. Moreover, the focus on pre-existing precarity has revealed the interconnections of economic insecurity and political and social insecurity more broadly. Thus, the pandemic does present a unique juncture in one essential respect: there is a growing awareness that nothing—the economy, education, health—works without suitable provision of care. If we are to follow Charlesworth’s exhortation to resist the allure of ‘crises’ and to refocus instead on an international law of the everyday, then there can surely be nothing more quotidian, yet essential, than care. The revaluation of care is a longstanding feminist project, in law and elsewhere, but without to date sustained and dedicated engagement by feminist international lawyers. If, as I propose, COVID-19 and its crisis framing has exposed the impoverishment of the Security Council as an agent for women’s rights and gender equality, and may yet inform a re-direction in feminist advocacy in the longer-term, then the refocusing and revaluing of care may yet provide the essential link between international law and the everyday.

## Footnotes

1 See United Nations Secretary-General (2020).

2 Ibid. See also, Guterres (2020).

3 See Gender Development Network (2020), Morgan (2020), Rigby (2020).

4 See Rees (2020).

5 See The NGO Working Group on Women Peace and Security (2020).

6 See Group of Women Leaders, Voices for Change and Inclusion (2020).

7 UNSC Res 1325 (2000) [on women and peace and security] S/RES/1325 (31 October 2000); UNSC Res 1820 (2008) [on acts of sexual violence against civilians in armed conflicts] S/RES/1820 (19 June 2008); UNSC Res 1888 (2009) [on acts of sexual violence against civilians in armed conflicts] S/RES/1888 (30 September 2009); UNSC Res 1889 (2009) [on women and peace and security] S/RES/1889 (5 October 2009); UNSC Res 1960 (2010) [on women and peace and security] S/RES/1960 (16 December 2010); UNSC Res 2106 (2013) [on sexual violence in armed conflict] S/RES/2106 (24 June 2013); UNSC Res 2122 (2013) [on women and peace and security] S/RES/2122 (18 October 2013); UNSC Res 2242 (2015) [on women and peace and security] S/RES/2242 (13 October 2015); UNSC Res 2467 (2019) [on conflict-related sexual violence] S/RES/2467 (23 April 2019); UNSC Res 2493 (2019) [on women and peace and security] S/RES/2493 (29 October 2019).

8 See Werner et al. (2020).

9 UNSC Res 2532 (2020) [on cessation of hostilities in the context of the coronavirus disease (COVID-19) pandemic] S/RES/2532 (1 July 2020).

10 Ibid, operative paragraphs 1 and 2.

11 Ibid, operative paragraph 3.

12 Ibid, operative paragraph 4.

13 Ibid, operative paragraph 5.

14 Ibid, operative paragraph 6.

15 Ibid, operative paragraph 7.

16 UNGA Res 74/270 (2020) ‘Global solidarity to fight the coronavirus disease 2019 (COVID-19)’.

17 UNSC Res 2532 (2020), operative paragraph 7.

18 See Office for National Statistics (2019).

19 UNSC Res 2532 (2020) [on cessation of hostilities in the context of the coronavirus disease (COVID-19) pandemic] S/RES/2532 (1 July 2020): 4, 6.

20 See further Davidson (2020).

21 See Women’s Policy Group Northern Ireland (2020).

22 Ibid, 7.
